# Exploring the Immunomodulatory Properties of Stem Cells in Combating COVID-19: Can We Expect More?

**DOI:** 10.3390/bioengineering10070803

**Published:** 2023-07-05

**Authors:** Panagiotis Mallis

**Affiliations:** 1 Hellenic Cord Blood Bank, Biomedical Research Foundation Academy of Athens, 4 Soranou Ephessiou Street, 115 27 Athens, Greece; pmallis@bioacademy.gr; Tel.: +30-6971616467; 2 Immunology Department & National Tissue Typing Center, General Hospital of Athens “Gennimatas”, 154 Mesogeion Ave., 115 27 Athens, Greece

**Keywords:** SARS-CoV-2, stem cells, COVID-19, IL-6, immune responses, mesenchymal stromal cells, HLA class I and II, next-generation sequencing

## Abstract

Since the first appearance of Severe Acute Respiratory Syndrome Coronavirus-2 (SARS-CoV-2) in December 2019, the disease has displayed a remarkable interindividual variability in the global population, resulting in different mortality and morbidity rates. Still, an effective cure against SARS-CoV-2 has not been developed, and therefore, alternative therapeutic protocols must also be evaluated. Considering that stem cells, especially Mesenchymal Stromal Cells (MSCs), are characterized by both regenerative and immunomodulatory properties and that their safety and tolerability have been investigated previously, these cells could potentially be applied against coronavirus disease 19 (COVID-19). In addition, an individual’s genetic background is further related to disease pathogenesis, especially rare Inborn Errors of Immunity (IEIs), autoantibodies against Interferon type I, and the presence of different Human Leukocyte Antigens (HLA) alleles, which are actively associated with protection or susceptibility in relation to SARS-CoV-2. Herein, the use of MSCs as a potential stem cell therapy will require a deep understanding of their immunomodulatory properties associated with their HLA alleles. In such a way, HLA-restricted MSC lines can be developed and applied precisely, offering more solutions to clinicians in attenuating the mortality of SARS-CoV-2.

## 1. Introduction

COVID-19 occurring due to SARS-CoV-2 was reported for the first time in December 2019 in Wuhan, China [1,2]. Since its first appearance, COVID-19 has been considered to be a pandemic disease. According to the World Health Organization (WHO), currently, more than 764 million cases and more than 6 million deaths have been reported globally [3]. By the time this article was prepared, COVID-19 had spread to more than 220 countries, with most cases having been identified in European countries (275,789,453), followed by the Western Pacific (202,604,225), Americas (192,187,133), South East Asia (61,021,914), Eastern Mediterranean (<23,340,000), and Africa (<9,000,000) [3].

The COVID-19 pandemic is a rapidly evolving infectious disease that is responsible for significant morbidity and mortality in the general population [4]. Moreover, COVID-19 comprises a heavy economic burden, where public and private finances have been negatively affected due to the enforcement of the lockdown [5]. Especially in developing countries, COVID-19 has led to important drawbacks in terms of their economic evolution, leading to work losses or salary reductions [5].

The virus spreads through various means, such as direct contact with infected patients, through droplets, or via aerosol transmission [6]. SARS-CoV-2 primarily infects the upper and then the lower respiratory system, causing mild or moderate symptoms, such as coughing, fever, fatigue, and general malaise [6,7]. Besides these symptoms, SARS-CoV-2 can significantly affect the function of multiple organs, such as the liver, kidney, intestines, and heart [6,7]. Furthermore, the pathogenetic mechanism of SARS-CoV-2 also involves moderate to severe manifestations in the host, such as the occurrence of pneumonia, accompanied by low oxygen levels of <90% or more serious lung tissue damage due to the presence of overstimulated immune cells and cytokine release syndrome (CRS). These events may lead to the admittance of critically ill COVID-19 patients into the intensive care unit (ICU) or may be related to other life-threatening issues [8]. Besides its global transmission and disease pathogenesis, COVID-19 has an average mortality rate of <6%; however, this rate is increased in elderly people > 65 years or patients suffering from important underlying disorders, including autoimmune and cardiovascular diseases and cancer [4,9]. 

Often, pandemics are responsible for the occurrence of strong selection in humans, and the variability and severity of COVID-19 among individuals are still under global investigation. This can be explained by the fact that interindividual genetic background may be related to resistance or susceptibility to SARS-CoV-2 transmission and pathogenesis. 

Besides the in-depth research concerning SARS-CoV-2 that has been conducted since the start of the outbreak, the scientific community has more to learn regarding the pathophysiological mechanisms of the disease and their association with human genetic and immunological backgrounds.

Indeed, genetic diversity between different ethnic groups, in combination with individuals’ characteristics, are considered to be major leading causes of the variability in terms of disease outcomes among humans [10]. 

The significant worldwide impact of the COVID-19 pandemic was translated into a comprehensive investigation and rapid development of therapeutic strategies designed to prevent its prevalence. Nowadays, accepted therapeutic protocols for efficiently combating SARS-CoV-2 involve (a) vaccination (mRNA vaccines), (b) monoclonal antibodies against viral proteins (e.g., bebtelovimab, sotrovimab, regdanvimab, bamlanivimab, etc.), (c) antiviral drugs (remdesivir, lopinavir), and (d) antibodies derived from convalescent plasma [11,12]. Among them, vaccination using mRNA products, viral proteins, or weakened strains is used globally, only reporting mild adverse reactions [11]. Accessing the database of clinical trials (www.clinicaltrials.gov (accessed on 8 June 2023)), more than 4000 trials searching for therapeutic protocols against SARS-CoV-2 are currently being performed, with some of them having passed to the next stage of the evaluation process. 

Among them, stem cells have also been proposed and evaluated as potential alternative COVID-19 therapeutics [13]. In contrast to the already presented therapeutic protocols, stem cells may represent an approach with only a few adverse reactions [13]. In the past, stem cells have been comprehensively evaluated concerning their regenerative and immunomodulatory properties. In addition, stem cells can originate from both autologous or allogeneic sources, while their isolation, expansion, and characterization represent a cost-effective, non-demanding task when compared to other therapeutic approaches [13].

In this editorial, the beneficial properties of stem cells in terms of combating SARS-CoV-2 will be summarized. The information provided here may be valuable for the scientific community in better understanding the biology of stem cells, which may serve as alternative therapeutic agents for better administration in human immune-related disorders.

## 2. SARS-CoV-2 Biology and Characteristics

Since the initial appearance of SARS-CoV-2, the original strain has undergone a great number of mutations, thus resulting in the identification of variants with differences in transmission and pathogenetic mechanisms [14]. Currently, the variant Omicron XBB 1.5 is characterized by the 87.9% predominance, while the XBB1.9.1 has the 4.6% predominance, nationwide [14,15].

SARS-CoV-2 is a positive-sense single-stranded RNA virus (+ssRNA) of *Betacoronavirus* genus group 2 and belongs to the *Coronaviridae* family [16]. The genome sequence of SARS-CoV-2 shares sequence similarity to the CoVs of the 2002–2003 outbreak [17]. Indeed, SARS-CoV-2 shares ~80% genome identity with SARS-CoV and ~50% with MERS-CoV. SARS-CoV-2 includes 14 open reading frames (ORFs), which mostly encode 16 structural proteins (nsp1-nsp16) [17,18]. The genome of SARS-CoV-2 contains genes responsible for the production of the accessory and the four structural proteins, including spike (S), envelope (E), membrane (M), and nucleocapsid (N) [19]. SARS-CoV-2 utilizes the S protein through a receptor-binding domain (RBD) to promote its entry into host cells. Specifically, the SARS-CoV-2 S protein binds to angiotensin-converting enzyme 2 (ACE2) [20,21]. The produced complex (S-ACE2) is further favored by the function of cathepsin L and the transmembrane protease serine 2 (TMPRSS2). Importantly, TMPRSS2 mediates the fusion of SARS-CoV-2 to the plasma membrane surface. Cathepsin L plays a significant role in viral entry by promoting SARS-CoV-2 entry in cells lacking TMPRSS2 [16,20,21]. Once the virus has entered the cells, ORF1a and ORF1b are initially translated into the viral replicase proteins, forming RNA-dependent RNA polymerase [22,23]. The resulting polyproteins, pp1a and pp1ab, are post-translationally processed, leading to the production of non-structural proteins (nsps), which are responsible for the viral replication and transcription complex. In this way, the rearrangement of the endoplasmic reticulum (ER) is performed to produce double-membrane vesicles (DMVs), representing a protective microenvironment for viral replication [23]. Specifically, within DMVs, the virus replicates its genomic and subgenomic mRNAs and further facilitates the production of accessory and viral structural proteins, which are responsible for the formation of viral particles [22]. The translated structural proteins are transited through the ER-to-Golgi intermediate compartment (ERGIC), interact with genomic RNAs and budded to the lumen of the secretory vesicular compartment, and the latter results in the exocytosis of the produced virions from the infected cells [23].

SARS-CoV-2 can also be transmitted to immune cells, such as macrophages, monocytes, and T and B cells; furthermore, viral transcripts have also been found in dendritic cells (DCs) [24]. SARS-CoV-2, besides its primary receptor, ACE2, can utilize additional receptors and co-receptors for its entry into the host’s cells, including C-lectin, such as DC-SIGN, L-SIGN, and ACRG-1 and transmembrane proteins, such as KREMEN1, Neuropilin-1, and AXL receptors. In addition, TMPRSS2 and cathepsin L are expressed in the immune cells, which are favored in terms of viral entry [25]. There is a growing body of evidence relying on autopsy reports, analyses of bronchoalveolar lavage fluid as well as in vitro conducted experiments, and these suggest that SARS-CoV-2 can replicate in cells of innate and adaptive immunity [24]. In such a way, macrophages infected with SARS-CoV-2 may be located in various organs, such as the lungs, spleen, lymph nodes, liver, heart, etc., promoting further viral transmission into the host. 

Cases of patients whose immune system cannot effectively combat and defeat the primary entry of the virus are related to great replication and the spreading of SARS-CoV-2 to various organs, leading to life-threatening events [26]. Indeed, critically ill SARS-CoV-2 patients need advanced hospitalization care and require ICU admission to decrease the possibility of life loss. Recent evidence suggests that infected macrophages and monocytes can lead to overactivated immune responses and CRS occurrence through either the release of inflammasomes or through pyroptosis by the infected cells [25]. The latter comprises lytic programmed cell death performed by the infected cells, leading to the release of damage-associated molecular patterns (DAMPs) and inflammatory cytokines, such as interleukin 1β (IL-1β), tumor necrosis factor-A (TNF-A), IL-3, and IL-6 [25].

The above mechanism is considered responsible for the recruitment and migration of DCs to the inflammation site. Activated DCs produce high levels of IL-1β, IL-2, IL-6, IL-8, interferon (IFN)-α/β, TNF-A, C-C motif chemokine 3 (CCL3), CCL5, CCL2, and IP-10, which further lead to the formation of an inflammatory microenvironment, stimulating T helper (Th)1, Th2 and Th17 and natural killer (NK) cells. The overactivation of these immune cells leads to increased IL-1B, IL-1RA, IL-7, IL-8, IL-9, TNF-A, fibroblast growth factor (FGF), transforming growth factor-β1 (TGF-β1), granulocyte–macrophage colony-stimulating factor (GM-CSF), IFN-γ, G-CSF, IP10, MCP1, MIP1A, platelet-derived growth factor (PDGF), TNF-α, vascular endothelial growth factor (VEGF), which represents the CRS phenomenon in critically ill COVID-19 patients [8,22,25]. 

Besides serious CRS, patients with severe SARS-CoV-2 infection also present with other manifestations, including thrombocytopenia, peripheral lymphopenia, respiratory failure due to lung damage, pneumonitis, and organ failure, which is highly related to loss of life [8,22,24].

## 3. Stem Cells and the Immune System

The proper management of COVID-19 still represents a great challenge; however, the promotion of vaccination programs and the combined use of advanced therapeutic protocols have proven to be lifesaving measures. Sill, on the other hand, some patients do not respond well to gold-standard treatments; therefore, alternative therapeutic strategies must be strongly considered.

It is widely known that stem cells, due to their properties, can be applied in patients with various disorders, attenuating their symptoms or severe manifestations [27]. Specifically, certain types of stem cells are important mediators of immune modulation and can effectively reverse virus-mediated tissue damage by exhibiting plasticity and differentiation potential toward desired cellular populations [28,29]. MSCs are multipotent stem cells of mesodermal origin, exerting both immunomodulatory and regenerative properties, and their safety and tolerability have already been evaluated in clinical trials of various human diseases [30]. 

MSCs are characterized by a spindle-shaped morphology and can be derived from different human tissue sources, such as the bone marrow (BM), adipose tissue (AT), Wharton’s jelly (WJ) tissue, placenta, amniotic fluid (AF), the stromal vascular fraction (SVF), etc. [30,31]. Based on their origin, MSCs exhibit different characteristics in terms of their proliferation potential, plasticity, and secretory and transcriptional profiles [30,31]. Additionally, fetal MSCs (derived from WJ tissue, amniotic fluid, or the placenta) are characterized by few non-inherent epigenetic and mutagenic changes, have longer telomeres, higher telomerase activity and better genome stability compared to adult MSCs (derived from BM, AT, or SVF) [30]. Lately, the International Society for Cell and Gene Therapy’s (ISCT) MSCs committee updated and provided advanced criteria for the proper characterization and determination of the MSCs [32,33]. Among them, the endogenous gene regulation program orchestrated by SOX2, OCT4, and NANOG must also be precisely determined to confirm the stemness of the cell population [34]. Indeed, NANOG is an important key stemness modulator; thus, it has been shown that stem cells lacking NANOG expression are committed to differentiation to other cell types [34]. 

Among other stem cells, MSCs share a unique relationship with the immune system, efficiently modulating overactivated immune responses [30,31]. Under specific stimuli, such as a tissue injury or pathogen entry, MSCs can migrate through a gradient chemokine-dependent manner to the inflamed region. In this way, MSCs express CD44 and ανβ1 integrin on their plasma membrane, thus performing “rolling” migration through the blood vessels upon reaching the injury site [30,35,36]. Then, MSCs can secrete specific trophic factors, such as TGF-β1, FGF, VEGF, interleukins (IL)-1a, IL-3, IL-6, and TNF-a, promoting the migration, activation, and proliferation of macrophages, DCs, NK, and T and B cells. Additionally, MSCs, upon stimulation with high levels of IFN-γ, TNF-α, IL-1 and IL-6, representing known inflammatory cues, can exert immunoregulatory properties through either direct contact (with the overactivated immune cells) or the production of soluble factors, such as nitric oxide (NO), indoleamine 2,3 dioxygenase (IDO), hepatocyte growth factor (HGF), human leukocyte antigen (HLA) g isoforms (G1-G7), galectins, prostaglandin E2, anti-inflammatory cytokines, such as IL-1Ra, IL-10, and IL-13, and micro-RNAs, including miR-21-5p, miR-142-3p, miR-223-3p, and miR-126-3p [30,31,36]. In the context of immune modulation, MSCs can lead to macrophage phenotype polarization from M1 to M2, inhibit DC and NK cell activation and lead to the programmed death of T and B cells. On the other hand, MSCs can promote the differentiation of CD4+ T cells into CD4+ CD25+ Foxp3 T regulatory cells and the production of tolerogenic DCs from CD34+ stem cells, favoring, even more, the modulation of overactivated immune responses [30,31,36].

Due to their function as “sensor” and “switcher”, MSCs are broadly used in a great number of clinical trials focusing on the proper administration of autoimmune diseases (such as multiple sclerosis, systemic lupus erythematosus, and Crohn’s disease), cancer and other immune-mediated human diseases, such as COVID-19 [30]. Moreover, MSCs have been shown to effectively suppress delayed-type hypersensitivity and graft-versus-host disease (GvHD) due to HLA compliance mismatch after hematopoietic stem cells (HSCs) or solid organ transplantation [30].

## 4. Stem Cells in Combating COVID-19

Due to the immunoregulatory properties of MSCs, these stem cells can be considered to be an alternative therapeutic strategy in critically ill COVID-19 patients, attenuating CRS and tissue damage. MSCs have been shown to tolerate the CRS induced by the pathogenetic mechanism of SARS-CoV-2 through the production of anti-inflammatory cytokines and other immunoregulatory soluble factors, as has been previously described [31,36,37]. MSCs may also assist in the prevention or shortage of the manifestations of long-COVID-19 syndrome [38]. Currently, numerous clinical trials utilizing either autologous, allogeneic, adult-, or fetal-derived MSCs against SARS-CoV-2 are underway with promising results [39,40,41]. However, to achieve the best outcome through the administration of MSCs, different factors that may induce variability in their immunoregulatory properties must be determined. For this reason, it has been considered that the variability in terms of the beneficial outcome of MSCs may be associated primarily with two factors: (a) cell source and (b) individual characteristics.

It has already been discussed that key differences regarding the stemness, proliferation, and differentiation potential exist between adult- and fetal-derived MSCs [30]. In addition, the individual’s health condition and medical history can be associated with specific mutations or epigenetic changes to the genome of the MSCs, mostly affecting their immunoregulatory and regenerative properties [30]. Therefore, MSCs should be appropriately evaluated using advanced methodologies, such as next-generation sequencing (NGS) analysis, mass cytometry, proteomic approaches, etc., to properly define their characteristics prior to infusion to patients.

On the other hand, individual characteristics also exhibit a central role in disease pathogenesis, explaining the variances in morbidity and mortality rates that occurred due to infectious diseases, such as SARS-CoV-2. Indeed, the introduction of SARS-CoV-2 to a naïve population worldwide resulted in clinical variability, from asymptomatic infections without any observed manifestations to mild and severe life-threatening manifestations. After the SARS-CoV-2 outbreak, the rapid identification of its genome helped us to better understand that interindividual discrepancy is associated with different disease outcomes [42]. However, we have more to learn about the association between COVID-19 and human genetic background, which can further lead to SARS-CoV-2 resistance or susceptibility. Evidence to enhance this consideration occurred by the fact that during the COVID-19 pandemic, all members of a family living in the same household were infected except one, showing promising resistance against SARS-CoV-2. In the past, there were also paradigms of other infectious diseases, such as tuberculosis, SARS, and MERS, showing that an individual’s genetic characteristics may differentially affect disease susceptibility from mild to severe manifestations [43,44,45]. 

Technological advances and, importantly, the wide application of NGS technology have allowed us to decipher an individual’s genetic background in-depth and associate it with underlining resistance against infectious disease. Indeed, rare IEIs have been shown to differentially affect the pathogenesis of viral infections, such as SARS-CoV-2 [44]. The COVID Human Genetic Effort (http://www.COVIDhge.com (accessed on 10 June 2023)) has already reported that at least 15% of cases of critically ill COVID-19 patients have been associated with IEIs of IFN type I [46]. Moreover, IEIs also concerning Toll-like receptor 3 (TLR3) and Interferon regulatory factor (IRF) 7 have been reported in previously healthy adults. These findings show that IFN type I is an indispensable factor that can control the pathogenesis and manifestations of SARS-CoV-2 [44]. Another important finding in the subsequent performed studies by the same consortium was the presence of preexisting neutralizing antibodies against IFN type I. More than 10% of critically ill COVID-19 patients had the aforementioned neutralizing antibodies [47,48,49]. In addition, patients older than 70 years old were characterized by a higher number of neutralizing antibodies against different proteins, such as interferons and cytokines of other soluble factors favoring the immune responses [44,47,49]. 

In addition to IEIs, an individual’s genetic prophylaxis against infectious disease is attributed due to other genetic factors. Indeed, the HLA system is currently associated with different human disorders such as autoimmune diseases, metabolic syndromes, and cancer [50]. HLA class I and II alleles are encoded by highly polymorphic genes and are responsible for pathogen recognition in promoting specific immune responses. Indeed, more than 30,000 HLA alleles, which encode approximately more than 18,000 proteins, have been identified and deposited in the IPD-IMG/HLA database (https://www.ebi.ac.uk/ipd/imgt/hla (accessed on 10 June 2023)) [50]. A great number of nucleotide substitutions, encoded by different HLA genes, concern the peptide-binding groove and their interaction site with the T cells (e.g., T cell receptors). To date, the literature has comprehensively described their strong association with (a) infectious diseases, such as HIV, HBV, H1V1, and HCV, (b) autoimmune diseases, such as multiple sclerosis, lupus erythematosus, Crohn’s disease, and (c) other human disorders, including cardiovascular disease, diabetes mellitus, and respiratory diseases [44]. Regarding COVID-19, the association of different HLA alleles with the prevalence, resistance, and severity of SARS-CoV-2 has also been reported [51]. In the context of immune responses against viral pathogens, an individual’s ability to exert an effective immune response can be associated with HLA alleles, which are primarily responsible for the presentation of antigenic epitopes CD8+ T and CD4+ T lymphocytes [51]. In genotyping analysis focused on the Italian population it was shown that the HLA haplotypes HLA-A*02:05-HLA-B*58:01, HLA-A*02:05-HLAC*07:01, HLA-A*02:05-HLA-B*58:01-HLA-C*07:01, HLA-A*02:05-HLA-B*18:01-HLA-DRB1*16:01, HLA-A*02:05-HLA-B*58:01- HLA-DRB1*03:01, HLA-A*02:05-HLA-B*58:01-HLA-C*07:01-DRB1*03:01, HLA-A*02:05-HLA-B*58:01-HLA-C*07:01-HLADRB1*16:01 have a protective role against SARS-CoV-2 [52]. On the other hand, susceptibility against SARS-CoV-2 is associated with haplotypes HLA-C*04:01-HLA-B*40:01-HLA-B*40:02, HLA-A30:02-HLA-B*14:02 and HLA-A30:02-HLA-C*08:02 [52]. In the same way, in a metanalysis study conducted in a Chinese population, the HLA alleles HLA-C*07:29, HLA-B*15:27 and HLA-B*54:01, HLA-B*56:01, and HLA-B*56:04 were found more frequently in critically ill COVID-19 patients [53]. Similarly, another study concerning the UK population showed that HLADRB1*15:01, HLA-DQB1*06:02, HLA-DRB1*10, and HLA-A*26 alleles are associated with enhanced SARS-CoV-2 susceptibility [54].

Accumulating evidence has shown that inflammatory stimuli due to high levels of IFN-γ, TNF-α, ΙL-6, or other factors, can lead to MSC activation and the expression of intracellular HLA-class II alleles at their plasma membrane [55]. In this way, MSCs can be involved in the mechanism of pathogen recognition and presentation to immune cells, thus enhancing both native and adaptive immune responses. Moreover, MSCs characterized by specific HLA haplotypes may be related positively or negatively to their immunoregulatory potential against specific infectious diseases. In addition, MSCs characterized by specific IEIs (concerning the IRF-7 and TLR-3) may be associated with key differences in their properties, affecting, in this way, their immunoregulatory and therapeutic potential against SARS-CoV-2 [56].

Ensuring the safe use of the MSCs requires good determination of their characteristics, such as their genetic background and secretory profile. Nowadays, utilizing state-of-the-art methodologies such as NGS and bioinformatic tools can yield more information regarding their genetic profile in terms of determining their HLA haplotypes and the presence of specific IEIs. Indeed, bioinformatic analytic tools have been developed to assist the scientific community in solving complicated biomedical issues. In our case, SARS-CoV-2 proteins such as S, E, M, and N were rapidly isolated and comprehensively studied, leading to the presentation of their nucleotide sequences to the scientific community worldwide. These key specific SARS-CoV-2 proteins can further be submitted in advanced bioinformatic analytic tools, such as NetMHCPan v.4.1, PickPocket, CD4Episcore, MHC-NP, MHCII-NP, and others, to better predict the affinity associations with HLA alleles [57]. Utilizing all of the available tools, specific MSC lines characterized by specific genetic backgrounds (considering their HLA haplotypes and IEIs) and secretory profiles can be isolated, expanded, and banked in order to be used based on a patient’s genetic background, ensuring proper disease administration and bringing precision medicine one step closer to clinical utility.

## 5. Concluding Remarks

In recent years, an enormous amount of data has been collected, showing the relationship between an individual’s genetic background and SARS-CoV-2 pathogenesis [46]. Therapeutic protocols, such as the use of antiviral factors (e.g., lopinavir, danoprevir, and nelfinavir), neutralizing antibodies (against IL-6 or IL-6 receptors, mAbs derived from convalescent plasma), or vaccination (mRNA, viral particles) have resulted in variable results in terms of protection against SARS-CoV-2. Moreover, current therapeutic protocols may have impaired potential in specific groups, such as elderly people, patients with autoimmune or chronic disorders, HIV-seropositive individuals, or people with preexisting severe medical conditions. In addition, the rapid appearance of new SARS-CoV-2 variants with varying immunoevasive properties may result in significant drawbacks in preparing virus-specific therapeutic protocols. Therefore, alternative strategies, such as the use of stem cells, either in an autologous or allogeneic state, should be strongly considered. Currently, the safety and tolerability of MSCs applied in various human disorders have been long evaluated through a great number of clinical trials [30].

In this way, an effective immunological strategy using stem cells should have two aims. The first aim should be the proper characterization of the properties of the stem cells, such as their genome stability, epigenetic profile, stem cell characteristics based on ISCT criteria, and secretory profile [32,33]. By acquiring these data, properly defined stem cells can be rapidly expanded under in vitro culturing conditions to great numbers, offering a potential pool of cells that can be immediately applied to patients. The second aim should be the consideration of the relationship between the IEIs, HLA alleles and SARS-CoV-2 prevalence. Analyzing stem cells using high-throughput methodologies offers a deep understanding and deciphering of their underlying cellular machinery in terms of combating SARS-CoV-2 or other pathogens. In this way, HLA-restricted stem cell lines may be developed and cryopreserved in cell and tissue banks, offering an alternative strategy against COVID-19 or other infectious diseases.

No specific drug effective against COVID-19 currently exists. Therefore, great efforts must be taken globally to produce available and safe therapeutic options. As part of this effort, our research group has also focused on the deep characterization of the properties of fetal- or adult-derived MSCs associated with the HLA class I and class II alleles. The collected data were analyzed through the use of advanced bioinformatic tools, obtaining significant information regarding their potential beneficial effect against SARS-CoV-2. Finally, the conclusions extracted due to the accomplishment of this effort may furnish new perspectives into the development of more precise therapeutic strategies with greater immunogenic potential against SARS-CoV-2 or other viral pathogens, reducing mortality rates and offering better weapons to clinicians.
